# Melatonin Supplementation Decreases Hypertrophic Obesity and Inflammation Induced by High-Fat Diet in Mice

**DOI:** 10.3389/fendo.2019.00750

**Published:** 2019-11-05

**Authors:** Talita da Silva Mendes de Farias, Maysa Mariana Cruz, Roberta Cavalcante da Cunha de Sa, Ilenia Severi, Jessica Perugini, Martina Senzacqua, Suzete Maria Cerutti, Antonio Giordano, Saverio Cinti, Maria Isabel Cardoso Alonso-Vale

**Affiliations:** ^1^Post-graduate Program in Chemical Biology, Institute of Environmental Sciences, Chemical and Pharmaceutical, Universidade Federal de São Paulo-UNIFESP, Diadema, Brazil; ^2^Department of Experimental and Clinical Medicine, University of Ancona (Politecnica Delle Marche), Ancona, Italy; ^3^Center of Obesity, University of Ancona (Politecnica Delle Marche), Ancona, Italy; ^4^Department of Biological Sciences, Institute of Environmental Sciences, Chemical and Pharmaceutical, Universidade Federal de São Paulo-UNIFESP, Diadema, Brazil

**Keywords:** subcutaneous fat, cytokines, inflammation, triacylglycerol, cholesterol, body weight reduction, CLS

## Abstract

Obesity results from critical periods of positive energy balance characterized by caloric intake greater than energy expenditure. This disbalance promotes adipose tissue dysfunction which is related to other comorbidities. Melatonin is a low-cost therapeutic agent and studies indicate that its use may improve obesity-related disorders. To evaluate if the melatonin is efficient in delaying or even blocking the damages caused by excessive ingestion of a high-fat diet (HFD) in mice, as well as improving the inflammatory profile triggered by obesity herein, male C57BL/6 mice of 8 weeks were induced to obesity by a HFD and treated for 10 weeks with melatonin. The results demonstrate that melatonin supplementation attenuated serum triglyceride levels and total and LDL cholesterol and prevented body mass gain through a decreased lipogenesis rate and increased lipolytic capacity in white adipocytes, with a concomitant increment in oxygen consumption and *Pgc1a* and *Prdm16* expression. Altogether, these effects prevented adipocyte hypertrophy caused by HFD and reflected in decreased adiposity. Finally, melatonin supplementation reduced the crown-like-structure (CLS) formation, characteristic of the inflammatory process by macrophage infiltration into white adipose tissue of obese subjects, as well as decreased the gene expression of inflammation-related factors, such as leptin and MCP1. Thus, the melatonin can be considered a potential therapeutic agent to attenuate the metabolic and inflammatory disorders triggered by obesity.

## Introduction

Obesity is a worldwide problem and represents a serious public health challenge for the 21'st century. In 2016, the World Health Organization (WHO, 2019), indicated that 1.9 billion adults over age 18 are overweight, of these, over 650 million were obese and about 3.4 million adults die each year due to co-morbidities associated with obesity such as hypertension, heart disease, dyslipidemia, fatty liver, type 2 diabetes, and some types of cancers. Obesity results from critical periods of positive energy balance characterized by caloric intake greater than energy expenditure, where the excess of calories from the diet are stored in white adipose tissue (WAT) in the form of triacylglycerols (TAG). The increase in fat mass can occur by two process: adipocyte hypertrophy or adipocyte hyperplasia (through *de novo* differentiation from progenitors) (1). It is known that adipocyte hypertrophy leads to morbid obesity (2, 3) characterized by the rapid growth of the fat depots through enlargement of existing fat cells, which is accompanied by a high degree of M1 macrophage infiltration, limited vessel development, and massive fibrosis (3). Considering these facts, such pathological expansion is associated with chronic inflammation and a WAT dysfunction.

WAT dysfunction is certainly one of the main causes of obesity-associated medical comorbidities, since this tissue is one of the first to develop inflammatory responses triggering the activation of the classical proinflammatory pathways, exacerbated infiltration of macrophages, neutrophils, lymphocytes, and a induction of a wide range pro-inflammatory mediators secretion (4, 5), which ultimately results in the development of systemic insulin resistance. A lot of therapeutic strategies are used to improve this condition triggered by this tissue dysfunction. According to some studies, the use of melatonin, a hormone produced by the pineal gland only in the night phase and responsible for the synchronization of innumerable physiological effects, is related to beneficial effects on the control of obesity and its complications (6–9).

Moreover, important melatonin effects in energy metabolism (10, 11) and insulin action on glucose and lipid metabolism have been showed, being many of this studies related to WAT from rodents, reported by our group (12–16). Additionally, chronobiological melatonin aspects and its interrelationship with cytokines produced by WAT such as leptin and adiponectin have been described (17, 18).

Another important effect described for melatonin was an anti-inflammatory action which occurs mainly due to its activity as a mitochondrial protector (19), by preventing insulin resistance (20, 21), as well as to present a role in the immune system, promoting a down-regulation of pro-inflammatory and an up-regulation of anti-inflammatory plasma cytokines in animal models of metabolic syndrome (22, 23).

All studies aforementioned reinforce the therapeutic potential for melatonin in treating obesity and its related complications. Considering that obesity leads to a dysfunction of the main metabolic processes of WAT (lipolysis, lipogenesis, and adipogenesis), the present study aims to evaluate if the melatonin is efficient in attenuating or even blocking the damages in WAT caused by the ingestion of a high-fat diet (HFD), as well as improving the inflammatory condition triggered by the HFD-induced obesity in mice.

## Materials and Methods

### Animals and Melatonin Supplementation

All procedures were approved by the Ethics Committee on Animal Use of the Federal University of São Paulo. Eight-week-old male C57BL/6 mice were maintained under controlled light-dark cycle (12 h:12 h lights cycle on at 0600), temperature of 24 ± 1°C and relative humidity 53 ± 2%. The mice were obtained from the Center for Development of Experimental Models (CEDEME), Federal University of São Paulo. They were randomly assigned into three groups: (a) control (low fat) diet (Control), (b) HFD (Obese), and (c) HFD supplemented with melatonin (Obese+Mel). Control diet contains 76% carbohydrate, 15% protein and 9% fat and a HFD contains 26% carbohydrate, 15% protein and 59% fat, in % kcal.

During obesity induction, the animals were supplemented with melatonin (1 mg/kg) in drinking water during the dark phase, daily, for 10 weeks. Body weight and food intake were measured weekly and the food and energy efficiency were calculated by the ratio of body weight gain (g) to food ingestion (g) or by ratio of body weight gain (g) to caloric intake (kcal). After 10 weeks of the experimental protocol, 12-h fasted mice were anesthetized with isoflurane and subjected to blood collection through puncturing the orbital plexus. The animals were euthanasied and tissues were removed after cervical dislocation. Adipose fat depots: ING (subcutaneous inguinal), EPI (epididymal), RP (retroperitoneal), and BAT (interscapular brown adipose tissue) were harvested and weighed. Then, ING depot was processed for RT-qPCR, adipocytes isolation and biological assays.

### Blood Measurements

Triacylglycerol, total cholesterol, LDL-cholesterol, and HDL-cholesterol levels were determined by colorimetric assays (Labtest Diagnostics, Lagoa Santa, MG, Brazil).

### Adipocyte Isolation

Adipocyte isolation was performed as previously described (24). Briefly, ING fat pads were diced in small fragments in a flask containing 4 mL of DMEM supplemented with HEPES (20 mM), glucose (5 mM), bovine serum albumin (BSA, 1%), and collagenase type II (1 mg/mL), pH 7.4 and incubated for 40 min at 37°C in an orbital shaker. Isolated adipocytes were filtered through a plastic mesh (150 μm) and washed three times in the same buffer without collagenase. Adipocytes were photographed under an optic microscope (×100 magnification) coupled to a microscope camera (AxioCam ERc5s; Zeiss, Oberkochen, Alemanha), and mean adipocyte volume (4/3 × π × *r*^3^) was determined by measuring 100 cells using AxioVision LE64 software.

### RNA Extraction and Quantitative Real-Time Polymerase Chain Reaction (qPCR)

Total RNA was extracted from ING depot, reverse transcribed, and destined for quantitative qPCR analysis as previously described (25). Analysis of real-time PCR data was performed using the $2_{\text{T}}^{-\Delta \Delta \text{C}}$ method. Data are expressed as the ratio between the expression of the target gene and housekeeping gene (18S gene). Primers used are presented in Supplementary Table 1.

### Lipolysis Measurement

Lipolysis was estimated as the rate of glycerol (Free Glycerol Determination Kit, Sigma) released from ING isolated adipocytes during 30 min of incubation (24). Results were expressed as nanomoles of glycerol per 10^6^ cells.

### Incorporation of [1-^14^C]-Palmitate Into Triacylglycerol

ING adipocytes were incubated in KRH (Krebs Ringer Hepes bicarbonate) buffer, pH 7.4, containing 1% BSA and 2 mM glucose plus palmitate (200 μM), saturated with a gas mixture of 95% O_2_ and 5% CO_2_, [1-^14^C]-Palmitate was then added to the buffer (1850 Bq/tube or well) and left for 2 h at 37°C. Cells were then washed three times with phosphate buffered saline (PBS) and Dole's reagent containing isopropanol:n-heptane:H_2_SO_4_ (4:1:0.25 vol/vol/vol) was added to the remaining reaction mixture for lipid extraction (24). The radioactivity trapped into TAG was determined using a β-counter (1450 LSC, Counter MicroBeta, Trilux; PerkinElmer). Results were expressed as nanomoles of FA per 10^6^ cells.

### Oxygen Consumption

Oxygen consumption rates in isolated cells were measured as an indication of mitochondrial respiratory activity. ING isolated cells from animals were gently re-suspended in KRH (pH 7.4) containing BSA (0.1%) and transferred to the oxygraph (OROBOROS Oxygraph-2 k). The oxygraph chambers were previously equilibrated with KRH containing BSA 0.1% at 37°C. Carbonyl cyanide m-chlorophenyl hydrazine (CCCP, 1 μM f.c.) was added as a positive control for maximal respiratory rate (uncoupling) determination. Oxygen consumption rates were normalized by cell number and expressed as % of the control (26).

### Perfusion, Fixation, Dehydration, and Embedding

For histochemical and immunohistochemical analysis, animals under deep anesthesia (100 mg/kg ketamine with 10 mg/kg xylazine) was perfused intracardially with 250 ml of 0.9% saline solution followed by 300 ml of fixation solution [4% paraformaldehyde in 0.1 M phosphate buffer (PBS), pH7.4]. The ING adipose depot was removed and pos-fixate for 12–15 h at 4°C. The tissue was washed in phosphate buffer to remove any residual fixative and was subsequently dehydrated with graded ethanol (from 50 to 100%), and cleared in a solvent (xylol) miscible with paraffin before impregnation at 55°C and finally embedded in paraffin.

### Light Microscopy and Morphometry

Serial paraffin sections 4 μm in thickness were obtained from ING tissue and mounted on slices. Some were stained with hematoxylin and eosin (H&E) to assess morphology; the others were used for immunohistochemical procedures (*n* = 6 for each procedure) (27). Adipocyte size was calculated as the mean adipocyte area of 300 random adipocytes (100 per section) from each depot of each mice using a drawing tablet and the Nikon Lucia Image software (version 4.61) of the morphometric program. Tissue sections were observed with a Nikon Eclipse E800 light microscope (Nikon Instruments, Firenze, Italy) using a 20X objective, and digital images were captured with a Nikon DXM 1220 camera.

### Immunohistochemical Analysis

Evaluation of macrophage infiltration and crown-like structure (CLS) density in the adipose tissue samples was performed by immunohistochemistry against MAC-2/galectin-3, a marker of activated macrophages, on paraffin-embedded slices. The primary antibody was a rat monoclonal anti-MAC-2 antibody (dilution 1:1,500; Cedarlane Laboratories, Burlington, Ontario, Canada). Immunohistochemistry and morphometrical analyses were performed according to Giordano et al. (27).

### Statistical Analysis

Data are presented as mean ± SEM. One-way ANOVA and Bonferroni or Tukey post-test were used for the comparison between groups. *Test-t* was used to verify the differences between Obese and Obese+Mel groups only. GraphPad Prism 5.1 software (GraphPad Software, Inc., San Diego, CA, USA) was used for analysis. The level of significance was set at *p* < 0.05.

## Results

**Figure 1:**
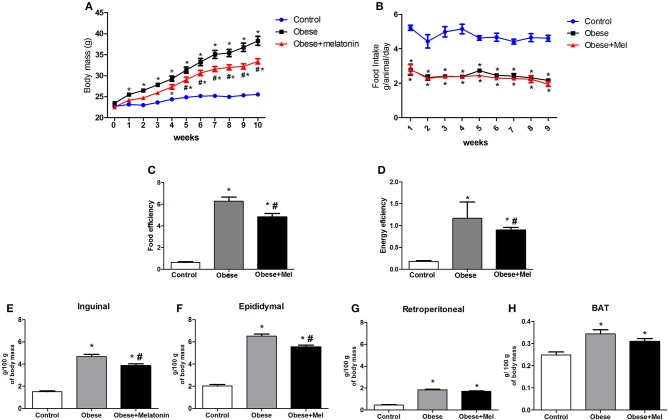
Effects of high-fat diet (HFD) and melatonin supplementation (Mel, 1 mg/ kg b.w., diluted in drinking water, daily, for 10 weeks) on body weight, food intake, and adiposity. Mice were fed with control diet (Control) or HFD (Obese), supplemented, or not with Mel (Obese+Mel). **(A)** Changes in body mass (g); **(B)** Food intake (g/ animal/ day); **(C)** Food efficiency body weight gain (g)/ food intake (g); **(D)** Energy efficiency body weight gain (g)/ caloric intake (kcal); Relative weight (mg/ 100 g body weight) of the **(E)** Subcutaneous adipose depot (inguinal -ING); **(F)** Visceral adipose depot (epididymal -EPI); **(G)** Visceral adipose depot (retroperitoneal -RP). **(H)** Interscapular brown adipose depot (BAT). Results were analyzed by two-way ANOVA and Bonferroni post-test. Values are mean ± SEM (*n* = 17–21). ^*^P < 0.05 vs. Control; ^#^*P* <.0.05 vs. Obese.

Body mass evolution, food ingestion, and the adipose depots weight of mice during the 10-weeks of the obesity-induction period are shown in the Figure 1 and both groups showed continued body weight increase. From week 2, the Obese group showed a weight gain around 15% (*p* < 0.05) higher than Control group and completed the experimental protocol (week 10) with a body mass ~49% higher (*p* < 0.05). However, the Obese+Mel group presented a significant increment on body weight when compared to Control only from week 4 and completed the experimental protocol (week 10) with the body mass ~28% higher (*p* < 0.05) than Control group, but 13% lower (*p* < 0.05) than Obese group (Figure 1A), although both groups presented the same pattern of food and fat ingestion (Figure 1B).

ING, EPI, RP, and interscapular BAT depots were removed and weighed, and statistical analysis showed that HFD increased the mass of these depots (3-fold, 3-fold, 4-fold, and 21%, respectively, *p* < 0.05, Figures 1E–H) compared to Control animals. Melatonin treatment significantly decreased the relative weight of the inguinal (~17%, Figure 1E) and epididymal (~15%, Figure 1F) WAT depots, compared to Obese group, but did not alter RP and BAT mass (Figures 1G,H).

Food efficiency (ratio between body mass gain and dietary intake), as well as energy efficiency (ratio of body mass gain and energy consumption) was also analyzed and the HFD (Obese) increased these parameters in ~9 and ~6-fold, respectively, compared to Control. Melatonin supplementation also significantly reduced (by 23%) both, food and energy efficiency (Figures 1C,D).

**Figure 2:**
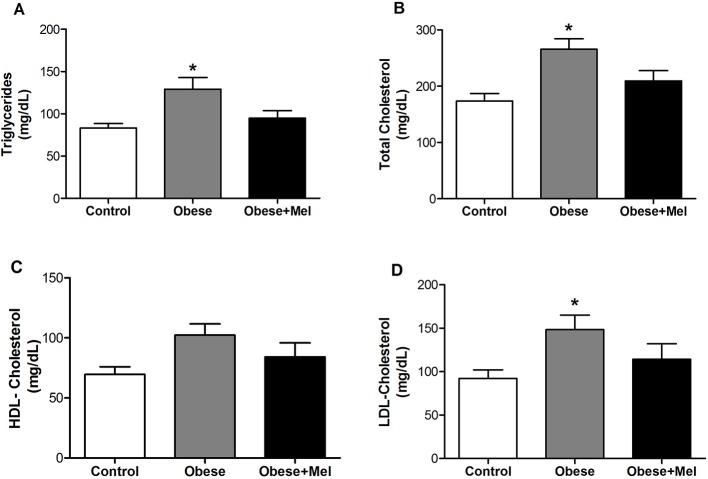
Effects of high-fat diet (HFD) and melatonin supplementation (Mel, 1 mg/ kg b.w., diluted in drinking water, daily, for 10 weeks) on serum levels of: **(A)** Triglycerides; **(B)** Total cholesterol; **(C)** HDL-cholesterol; and **(D)** LDL-cholesterol. Mice were fed with control diet (Control) or HFD (Obese), supplemented, or not with Mel (Obese+Mel). Results were analyzed by one-way ANOVA and Tukey post-test. Values are Mean ± SEM (*n* = 10–12). ^*^P < 0.05 vs. Control.

An significant increase in plasma total cholesterol (by 53%, Figure 2A), LDL -cholesterol (by 60%, Figure 2B) and triglycerides (by 52%, Figure 2D) was observed in Obese group. However, melatonin supplementation prevented these increment by 23, 28, and 25% for serum triglycerides, total cholesterol and LDL cholesterol, respectively, *p* < 0.05). Plasma HDL-cholesterol did not show any difference between the groups (Figure 2C).

**Figure 3:**
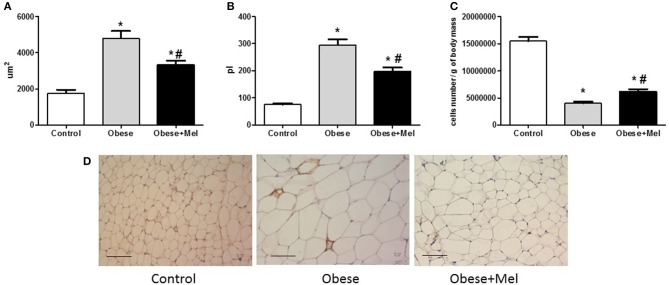
Effects of high-fat diet (HFD) and melatonin supplementation (Mel, 1 mg/ kg b.w., diluted in drinking water, daily, for 10 weeks) on ING adipocyte morphometry. **(A)** Adipocyte area of ING adipose depot; **(B)** Adipocyte volume; **(C)** ING cellularity; **(D)** Hematoxylin and eosin (H&E) staining. Mice were fed with control diet (Control) or HFD (Obese), supplemented, or not with Mel (Obese+Mel). Results were analyzed by one-way ANOVA and Tukey post-test. Values are mean ± SEM (*n* = 17–21 to adipocytes volume and *n* = 6 to histological analysis). Bar = 100 μm. ^*^P < 0.05 vs. Control; ^#^*P* < 0.05 vs. Obese.

Figure 3 presents the morphological study of adipocytes in the inguinal region. Mice consuming the HFD (Obese group) presented a significant hypertrophy of the fat cells, increasing its area (~1.5-fold) and volume (~2.3-fold) as compared to the Control group. The supplementation with melatonin attenuated this effect, since it decreased the area (~31%, *p* < 0.05) and the volume (~32%, *p* < 0.05) of the cells (Figures 3A,B, respectively), when compared to Obese group. These findings were confirmed by the histochemical. H&E-stained of ING longitudinal sections (Figure 3D). Melatonin also attenuated (~20%, *p* < 0.05) the effect of HFD on the marked drop (~70%) in the cellularity of this depot (Figure 3C).

**Figure 4:**
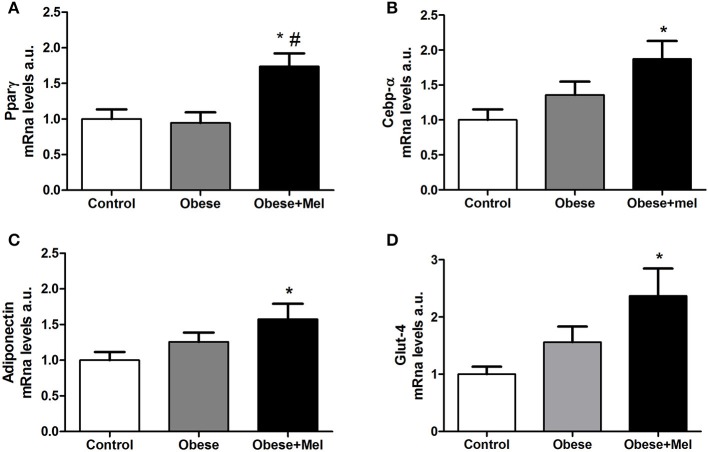
Effects of high-fat diet (HFD) and melatonin supplementation (Mel, 1 mg/ kg b.w., diluted in drinking water, daily, for 10 weeks) on mRNA levels of genes related to adipogenesis expressed ING adipose tissue. **(A)** mRNA levels of *Ppargama*; **(B)** mRNA levels of *C/ebpalfa*; **(C)** mRNA levels of A*diponectin*; **(D)** mRNA levels of *Glut-4*. *18S* was used as the housekeeping gene. Mice were fed with control diet (Control) or HFD (Obese), supplemented, or not with Mel (Obese+Mel). Results were analyzed by one-way ANOVA and Tukey post-test. Values are mean ± SEM (*n* = 9–13). ^*^P < 0,05 vs. Control; ^#^*P* < 0,05 vs. Obese.

Thus, we next evaluated the expression of CCAAT/enhancer-binding protein alfa (*C/ebpalfa*) and Peroxisome proliferator-activated receptor gamma (*Ppargama*), the major regulators of early adipogenesis. It was found a significant increase in gene expression of both transcription factors in the Obese+Mel group compared to Control (by 73 and 66% for *Ppargama* and *C/ebpalfa*, respectively) and Obese groups (by 84 and 40% for *Ppargama* and *C/ebpalfa*, respectively; Figures 4A,B).

C/EBPα and PPARγ are also required to maintain the differentiated state of mature adipocytes and insulin sensitivity, by activated PPARγ target genes such as adiponectin and Glucose transporter-4 (*Glut-4*), that are late markers of adipocyte differentiation. Corroborating the *Ppargama* and *C/ebpalfa* increase findings in obese animals supplemented with melatonin, the genes that encode Adiponectin and Glut-4 were also increased in the inguinal WAT of Obese+Mel group compared to the Control group (57% and by 2.3-fold, respectively; Figures 4C,D).

**Figure 5:**
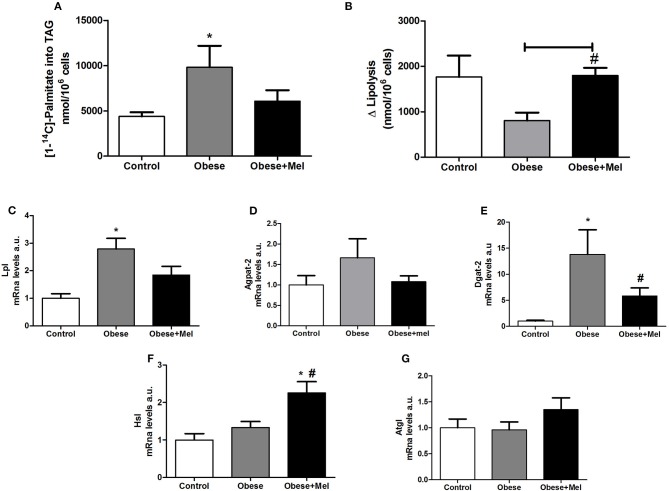
Effects of high-fat diet (HFD) and melatonin supplementation (Mel, 1 mg/ kg b.w., diluted in drinking water, daily, for 10 weeks) on lipogenesis and lipolysis in ING adipocytes. **(A)** [1^−14^]- palmitate incorporation into TGA (nanomoles of incorporated [1^−14^C]- palmitate per 10^6^ cells); **(B)** Lipolytic capacity; **(C)** mRNA levels of *Lpl*; **(D)** mRNA levels of *Agpat-2;* **(E)** mRNA levels of *Dgat-2;* **(F)** mRNA levels of *Hsl*; **(G)** mRNA levels of *Atgl*. Mice were fed with control diet (Control) or HFD (Obese), supplemented, or not with Mel (Obese+Mel). Results were analyzed by one-way ANOVA and Tukey post-test. Values are Mean ± SEM (*n* = 6–8 to metabolic activities and *n* = 9–13 to gene analysis). ^*^P < 0.05 vs. Control; ^#^*P* < 0.05 vs. Obese.

To investigate whether melatonin influence TAG metabolism in adipocytes from obese mice induced by HFD, the lipogenesis process was evaluated through studying the incorporation of fatty acids into TAG in inguinal isolated adipocytes. There was a significant increase (by 2.2-fold) in palmitate incorporation in the cells from obese animals, and this effect was partially reversed by melatonin supplementation for 10 weeks (reduction of 38% compared to Obese group; Figure 5A). Furthermore, the increase in lipogenesis induced by HFD was associated with significant upregulation in the mRNA levels of the lipogenic enzymes Lipoptotein lipase *(Lpl)* (~2.7-fold, Figure 5C) and Acyl CoA:diacylglycerol Acyltransferase 2 *(Dgat2)* (~13-fold), and partial reversion by melatonin supplementation, since a significant decrease in *Dgat2* gene expression was observed in the group that received melatonin (Obese+Mel) compared to Obese group (Figure 5E), but no difference was seen in relation to Control group. The 1-acylglycerol-3-phosphate O-acyltransferase 2 (*Agpat-2*) analysis did not present statistical differences between groups (Figure 5D).

In contrast to lipogenesis, mice receiving HFD presented a significant decrease in lipolytic capacity (~54%, *p* < 0.05) measured by the increment (delta) over basal, after isoproterenol-stimulated lipolysis in isolated adipocytes (Figure 5B). Melatonin supplementation completely prevented this fall. Furthermore, the melatonin effect in lipolysis was associated with significant upregulation in the mRNA levels of the lipase Hormone-Sensitive Lipase (*Hsl*) (~81%, *p* < 0.05; Figure 5F), but not Adipose triglyceride lipase (*Atgl*) (Figure 5G).

**Figure 6:**
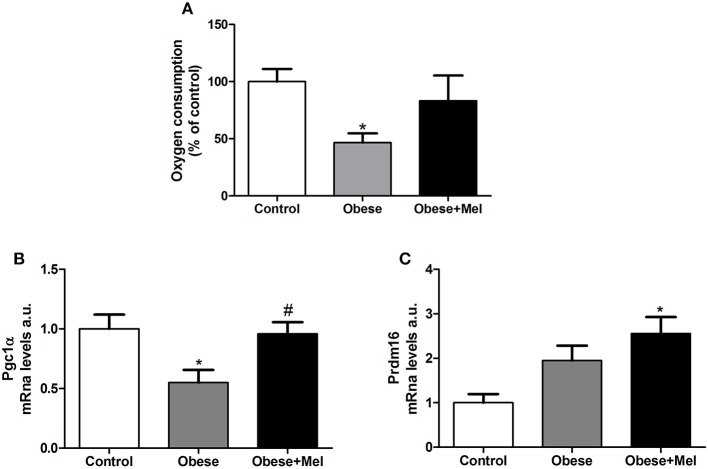
Effects of high-fat diet (HFD) and melatonin supplementation (Mel, 1 mg/ kg b.w., diluted in drinking water, daily, for 10 weeks) on oxygen consumption in ING adipocytes. **(A)** Oxygen consumption; **(B)** mRNA levels of *Pgc1alfa*; **(C)** mRNA levels of *Prdm16*. Mice were fed with control diet (Control) or HFD (Obese), supplemented, or not with Mel (Obese+Mel). Results were analyzed by one-way ANOVA and Tukey post-test. Results are presented as means ± SEM (*n* = 8–10). ^*^P < 0.05 vs. Control; ^#^*P* < 0.05 vs. Obese.

Figure 6 shows the oxygen consumption rate by isolated inguinal adipocytes. Melatonin supplementation partially reversed (~77%) the decrease (54%, *p* < 0.05) in oxygen consumption triggered by obesity (Figure 6A). The gene expression of the Peroxisome Proliferator-Activated Receptor Gamma Coativator 1-alpha (*Pgc1alfa*) and PR-domain containing 16 *(Prdm16)*, both transcriptional co-regulators that boost the increase of mitochondrial genes expression, were next evaluated. Melatonin supplementation completely prevented the drop (by 50%, *p* < 0.05, Figure 6B) in *Pgc1alfa* expression observed in non-supplemented Obese group. Melatonin also increased *Prdm16* expression (2.5-fold compared to Control group, Figure 6C).

**Figure 7:**
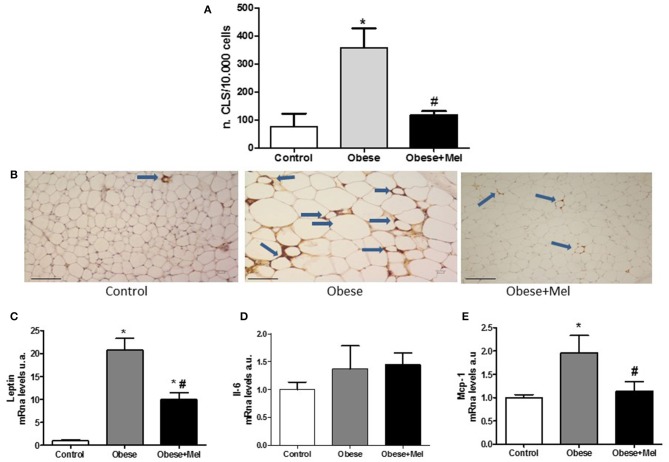
Effects of high-fat diet (HFD) and melatonin supplementation (Mel, 1 mg/ kg b.w., diluted in drinking water, daily, for 10 weeks) on inflammation in the ING depot. Mice were fed with control diet (Control) or HFD (Obese), supplemented, or not with Mel (Obese+Mel). **(A)** Immunohistochemistry analysis [number of crown-like structures (CLS)/ 10.000 adipocytes] using Mac-2 marker (*n* = 6). White adipose tissue (WAT) macrophages localize to CLS around individual adipocytes. **(B)** Light microscopy of inguinal WAT of Control, Obese, and Obese+Mel mice showing MAC-2 immunoreactive macrophages (arrows in brown color). Bar = 100 μm; **(C)** mRNA levels of *Leptin* in ING depot; **(D)** mRNA levels of *Il-6* in ING depot; **(E)** mRNA levels of *Mcp-1* in ING depot. Results were analyzed by one-way ANOVA and Tukey post-test. Values are mean ± SEM (*n* = 9–13). ^*^P < 0.05 vs. Control; ^#^*P* < 0.05 vs. Obese.

Obesity is also characterized by chronic low-grade inflammation and macrophage infiltration into WAT is implicated in the metabolic complications. Once active, macrophages aggregate and form the so-called crown-like-structure (CLS). In order to visualize these structures, we performed an immunohistochemistry analysis with the galectin-3 (also known as Mac-2 marker). The galectin-3 is a lectin expressed in activated macrophages which is related to mediate the inflammatory and phagocytic responses of macrophages. The analysis revealed that melatonin supplementation decreased ~2.6-fold (*p* < 0.05) the presence of these structures in the subcutaneous depot, compared to Obese group (Figures 7A,B).

In addition, genes that encode important cytokines involved in inflammation, such as leptin, interleukin-6 (*Il-6*), and monocyte chemoattractant-1(*Mcp-1*), were also evaluated. It was observed that both the *Leptin* and *Mcp-1* expression were significantly increased in mice receiving HFD (20-fold and 95%, respectively). Melatonin supplementation reversed the effect of HFD on the marked rise in L*eptin* and completely reversed *Mcp-1* increase (*p* < 0.05; Figures 7C,E). Regarding *Il-6* mRNA expression, no differences were observed between the groups (Figure 7D).

## Discussion

Herein, we investigated the repercussions of melatonin supplementation in WAT of obese mice induced by a HFD. Our data showed that melatonin prevents the body mass gain that corroborates the lower weight of inguinal and epididymal fat depots, as well as the smaller volume and area of its adipocytes. Melatonin also reduced the lipogenesis and acted to increase the lipolytic capacity and oxygen rate consumption of adipocytes from the inguinal fat. Furthermore, this hormone reduced CLS formation which is characteristic of obesity, showing that in addition to its metabolic effects, melatonin acts to reduce inflammation, that was confirmed by the lower expression of pro-inflammatory cytokines in WAT, such as leptin and Mcp-1.

Obesity triggered by the ingestion of a HFD may be a consequence of desynchronization in the biological rhythms of important metabolic processes (28, 29). The supplementation with melatonin (1 mg / kg) exclusively at night prevented or even partially reversed the changes observed in the obese phenotype. Thus, it is possible that melatonin acting on the synchronization of clock genes is preventing the desynchronization generated by the HFD intake. In addition, some studies show that melatonin, being a potent antioxidant, could improve inflammation by acting as a scavenger of reactive oxygen and nitrogen species (30). It is important to emphasize that the doses offered to the animals in other works studying obesity and inflammation ranged from 10 to 100 mg/kg, i.e., 10–100x more than we offered to the animals. We opted for this dose as it is considered a more physiological dose from a metabolic point of view. Anyway, even offering that small amount, we have already seen these beneficial effects.

Melatonin effect on body weight/fat mass was also described in others studies (31, 32). Using different animal models (ob/ob mice or Sprague Dawley rats), these authors observed that melatonin supplementation by gavage (30 mg/kg for 3 weeks) or in drinking water (100 mg/kg for 8 weeks) decreased ~5% the total body mass and the weight of visceral and subcutaneous fat depots. In another study, melatonin (4 mg/kg), did not affect body mass of obese animals, despite having generated a cardioprotective effect (33). These controversial data may be due to the different concentrations and methods of melatonin administration.

Associated to a body mass reduction, a preventive effect exerted by melatonin on serum triglycerides, total cholesterol and LDL-cholesterol levels due to HFD was herein observed. Hoyos et al. (34), also found a reduction in triglycerides and LDL levels and an increase in HDL serum levels in rats fed with cholesterol-enriched diet treated with melatonin. In another study (35), it was observed a reduction in body mass, triglycerides levels, cholesterol and LDL in rabbits with HFD-induced obesity treated with melatonin (1 mg/kg subcutaneous) for 4 weeks.

An attenuation of serum triglycerides and cholesterol levels has been attributed to the antioxidant action of melatonin, which in turn, reduces the effects caused by oxidative stress (36). It is important to note that dyslipidemia is associated with an increase risk of cardiovascular disease, once the obese and/or diabetic individuals showed a high plasma concentrations of total cholesterol and LDL-cholesterol (37). Thus, the attenuation of serum triglyceride levels, total cholesterol, and LDL cholesterol observed in animals treated with melatonin suggests a role in atherosclerosis prevention, one of the serious complications of obesity.

Corroborating the lower body mass gain observed in the melatonin group, the masses of both ING and EPI depots were reduced indicating that melatonin is able to influence the adiposity by preventing cell hypertrophy triggered by HFD. Interestingly, it was shown that only 24–48 h of exposure to a HFD is enough to cause an abrupt increase in adipocyte size (38). Different from ING and EPI, RP, and BAT are smaller adipose depots, thus, it is possible that the subtle effect of melatonin reducing adiposity was not sufficient to reflect on a relevant statistical analysis. Additionally, it is believed that the high-fat diet causes the process of tissue "whitening” in the brown adipose tissue. Therefore, melatonin supplementation for 10 weeks was not enough to completely block this process, despite the gross mass of this tissue in the supplemented animals to be smaller.

In spite of adiposity and hypertrophy prevention effect exerted by melatonin, we observed that the number of cells in ING depot was increased in obese+Mel when compared to obese animals (that present a significant decrease in adipocytes number) and, concomitantly to this effect, a significant increase in the expression of *Ppargama*, the master regulator of adipogenesis (39), was also observed. Although the role of melatonin in the adipogenesis process is not clear, a study using 3T3-L1 cells showed that melatonin stimulates the differentiation of these cells by increasing the expression of PPAR-γ and CEBP-α (40), whose data corroborates our findings.

The literature reports a subgroup of obese individuals classified as metabolically healthy but obese (MHO), that seems to be more resistant to the development of obesity-associated metabolic disorders. Despite the excess of body fat, these individuals exhibit a normal insulin sensitivity, arterial pressure and a favorable lipid, hormonal, inflammatory, hepatic, and immunological profile (41). Smaller and more numerous adipocytes are markers of a healthy obesity indicating that WAT expansion is promoted by increased cell number. For this reason, adipocyte differentiation is now accepted to be a potent strategy to allow for healthy WAT expansion and to prevent the development of hypertrophic obesity, an independent risk factor for the development of type 2 diabetes (2). Adipogenesis in subcutaneos stromal cells is markedly reduced in hypertrophic obesity and the degree of impairment in this process is positively correlated with adipose cell size (42).

In fact, it has been suggested that the damage in the ability to recruit and differentiate new subcutaneous adipose precursor cells is the cause of the hypertrophic obesity (43, 44). Once committed, preadipocytes can undergo the adipogenic program leading to activation of the dominant adipose regulator PPAR-γ as well as the C/EBP proteins (39, 45). Newly differentiated adipose cells secrets more adiponectin an important adipose-derived insulin-sensitizing hormone, one of the best predictors of insulin sensitivity and marker of adipogenesis. However, the serum levels of adiponectin drop considerably in hypertrophic (pathological) obesity (46). Thus, based on our data about hypertrophy and hyperplasia of the fat cells, we can infer that melatonin may act as a blocker agent in the development of pathological obesity, since the formation, and presence of smaller and more numerous adipocytes brings benefits to the individual, for example, expressing more adiponectin, as we observed in this study (Figure 4C).

Also, studies indicate that the improvement and increase in mitochondrial biogenesis is associated with adipocytes differentiation (47). Moreover, it has been demonstrated that mitochondrial function is damaged under conditions of type 2 diabetes and morbid obesity, indicating that under these conditions there is a reduction of total oxygen consumption rates (48), suggesting a decrease in mitochondrial oxidative activity. As observed here, the animals consuming a HFD showed a marked decrease in oxygen consumption. However, those supplemented with melatonin partially recovered this rate, approaching the levels observed in animals receiving a control diet. It is known that melatonin acts on mitochondria because is a potent scavenger of ROS (49) for this reason the improvement observed in this function could be given to the protective effect of melatonin by preventing oxidative stress induced by obesity (50). Other studies have indicated that melatonin could increase mitochondrial biogenesis (51) and inhibit apoptosis (52). The key gene for the mitochondrial biogenesis activation is *Pgc1-*α and can be transactivated by *Prdm16*, a gene that is intimately involved with the brown adipocytes differentiation (53). Our results point to this interrelation, since we observed a significant increase in the expression of both genes. Moreover, it is known that PPAR-γ (also increased in this study by the melatonin treatment) is also important to the thermogenic function and brown adipocytes differentiation (54, 55).

In addition to adipogenesis, adiposity is regulated by two fundamental metabolic processes in adipocytes, lipogenesis and lipolysis. Lipogenesis, an anabolic pathway responsible for the accumulation of TGAs, is also regulated by melatonin. Studies have shown that this neurohormone is able to inhibit the lipogenesis in the liver of hamsters (56) as well as promote a down-regulation on the expression of SREBP-1 (sterol regulatory element binding protein 1*), Fas (fatty acid synthase)*, and Scd1 (stearoyl-CoA desaturase-1) in HepG2 cells (57). In visceral adipocytes, melatonin increased *Fas* expression (13). Here, we demonstrated a decrease of ~40% in the incorporation of fatty acids into TAG in subcutaneous (ING) cells from obese mice receiving melatonin, as well as a reduction in the expression of *Lpl* and *Dgat2*, both important lipogenic enzymes (58). The analysis of *Agpat-2* gene expression, another enzyme involved with triglycerides biosynthesis, reveled a decrease of ~35% in its expression in the Obese+Mel group. Thus, melatonin was able to limit the triglycerides synthesis in ING adipocytes.

On the other hand, lipolysis, process by which occurs the lipid breakdown from adipocytes, generates free fatty acids and glycerol release and is important to WAT mass regulation (59). Lipolysis is mainly activated by catecholamines acting through its beta-adrenoreceptor and is strongly regulated by the hormone-sensitive lipase (HSL) enzyme. Studies have shown that melatonin, at different concentrations, is able to stimulating lipolysis through HSL and pHSL protein up-regulation, besides regulating the gene expression of *Atgl* and *Perilipin* (60). It was also shown that the melatonin can promote lipolysis of intramuscular fat by activating protein kinase A and the signaling of extracellular signal-regulated kinases 1/2 (61). Herein, it was observed that in addition to the increased lipolysis, melatonin stimulated the *Hsl* gene expression. Since the lipolysis activity is highly linked to the thermogenic process (62), the increase in the lipolytic capacity observed in Obese+Mel group reinforces the hypothesis that melatonin stimulates the thermogenesis as a consequence of an increment in the browning process. Although browning was not approached here, it was demonstrated in another work that melatonin (10 mg/kg/days) for 6 weeks was efficient in promoting browning in subcutaneos adipose depot of Zucker diabetic fatty rats (63).

In addition to its important metabolic function, WAT also secretes a variety of adipokines with pro- and anti-inflammatory characteristics (64). In obesity condition, it is observed a disbalance on this secretion, where a greater release of pro-inflammatory cytokines (Leptin, IL-6, TNF-α among others) is seen in detriment to anti-inflammatory cytokines (Adiponectin and IL-10). Our results indicates that HFD was effective in increasing the expression of *leptin, Il-6*, and *Mcp-1* and that melatonin supplementation significantly prevented the *leptin* and *Mcp-1* increase demonstrating the potent anti-inflammatory action of melatonin. As we did not perform the measures in a visceral adipose depot, but in subcutaneous, that is described to produce and release less IL-6 (24, 65), the statistical analysis was not able to demonstrate significant difference between the three groups.

The increase in adiposity also leads to a lower ability to expand the capillary network surrounding the adipocytes, resulting in hypoxia and cellular necrosis, which contributes to increasing inflammatory cell infiltrate (66). The macrophage infiltration into WAT is implicated in the metabolic complications of this comorbidity. Once the macrophages are active, using the Mac-2 marker, it is possible to visualize a set of structures called as CLS, which correspond on macrophages that individually surround each adipocyte during an inflammatory process (64, 67). As observed, melatonin decreased ~2.6-fold the presence of these structures in the subcutaneous depot of the obese animals, confirming that this neurohormone can attenuate the inflammatory framework. Herein, we describe for the first time the action of melatonin decreasing the CLS formation characteristic of the inflammatory process by macrophage infiltration in WAT of obese mice, induced by a HFD. This result corroborate the findings in isolated myofibroblasts induced to inflammation by leptin, where melatonin (1 μM) was able to reduce the increase of galectin-3 protein expression in these cells (68).

Taken together, our study provide further evidences proving the beneficial functions of melatonin on the obesity condition. More studies are necessary to understand the pathways by which melatonin acts to prevent and ameliorate the dysfunctions caused by increased adiposity in mice on the HFD. The major findings of our study is that the melatonin supplementation prevents the deleterious effects caused by the excessive intake of a hyperlipidic diet, and can be considered a potential therapeutic agent to attenuate the metabolic and inflammatory disorders triggered by obesity. This work provides important evidence concerning the effectiveness of a low-cost hormone to prevent or treat the obesity-related conditions, which causes thousands of deaths annually and burdens the public coffers.

## Data Availability Statement

All datasets generated for this study are included in the article/Supplementary Material.

## Ethics Statement

The animal study was reviewed and approved by Ethics Committee on Animal Use—UNIFESP number 5998280515.

## Author Contributions

TF: concept/design, design of experiments, acquisition of data, data analysis/interpretation, drafting, and revision of the manuscript. MC, RS, IS, JP, MS, and SMC: acquisition of data and revision of the manuscript. AG and SC: data analysis/interpretation and revision of the manuscript. MA-V: concept/design, data analysis/interpretation, drafting of the manuscript, revision of the manuscript, and approval of the article.

### Conflict of Interest

The authors declare that the research was conducted in the absence of any commercial or financial relationships that could be construed as a potential conflict of interest.
