# Supplementary material for: Melatonin Supplementation Decreases Hypertrophic Obesity and Inflammation Induced by High-Fat Diet in Mice
Source: Front Endocrinol (Lausanne). 2019 Nov 5;10:750. doi: 10.3389/fendo.2019.00750 (PMC6848267; doi:10.3389/fendo.2019.00750)
Supplement: Supplementary file 1 [file Data_Sheet_1.PDF]

Table 1: Sense e antisense primers sequences used for real-time quantitative PCR.

Gene	5’ Primer (5’-3’) - *Sense*	3’ Primer (5’-3’) – *Antisense*
18s	GGCCGTTCTTAGTTGGTGGAGCG	CTGAACGCCACTTGTCCCTC
Pgc1alfa	ATCTACTGCCTGGGGACCTT	ATGTGTCGCCTTCTTGCTCT
Prdm16	CAGCACGGTGAAGCCATTC	GCGTGCATCCGCTTGTG
Atgl	GGTCCTCTGCATCCCTCCTT	CTGTCCTGAGGGAGATGTC
Hsl	GGGAGGGCCTCAGCGTTCTCACA	ATAGCACGGAGCTGGGTGAGGG
Lpl	GGCCAGATTCATCAACTGGAT	GCTCCAAGGCTGTACCCTAAG
Glut-4	CATTCCCTGGTTCATTGTGG	GAAGACGTAAGGACCCATAGC
Leptin	CATCTGCTGGCCTTCTCCAA	ATCCAGGCTCTCTGGCTTCTG
Adiponectin	GCAGAGATGGCACTCCTGGA	CCCTTCAGCTCCTGTCATTCC
C/ebp-alfa	CGCAAGAGCCGAGATAAAGC	CAGTTCACGGCTCAGCTGTTC
Ppar-gama2	GCATCAGGCTTCCACTATGGA	AAGGCACTTCTGAAACCGACA
Mcp1	GCCCCACTCACCTGCTGCTACT	CCTGCTGCTGGTGATCCTCTTGT
Il-6	TTCTCTGGGAAATCGTGGAAA	TCAGAATTGCCATTGCACAAC
Agpat-2	CAGCCAGGTTCTACGCCAAG	TGATGCTCATGTTATCCACGGT
Dgat-2	GAAGCTGCCCGCAGCGAAAA	TCTTGGGCGTGTTCCAGTCAA

18s, 18s ribosomal RNA; Pgc1-alfa, Peroxisome Proliferator-Activated Receptor Gamma Coativator 1-alpha; Prdm16, PR-domain containing 16; Atgl, Adipose triglyceride lipase; Hsl, Hormone-sensitive lipase; Lpl, Lipoprotein lipase; Glut-4, Glucose- transporter-4; C/ebp-alfa, CCAAT/enhancer-binding protein alfa; Ppar-gama, Peroxisome proliferator-activated receptor gamma; Mcp-1, monocyte chemoattractant-1; Tnf-alfa, Il-6, interleukin-6; Agpat-2, 1-acylglycerol-3-phosphate O-acyltransferase 2; Dgat2, Acyl CoA:diacylglycerol Acyltransferase 2.
